# Phase 2 clinical trial of 5-azacitidine, valproic acid, and all-trans retinoic acid in patients with high-risk acute myeloid leukemia or myelodysplastic syndrome

**DOI:** 10.18632/oncotarget.106

**Published:** 2010-05-19

**Authors:** Emmanuel Raffoux, Audrey Cras, Christian Recher, Pierre-Yves Boëlle, Adrienne de Labarthe, Pascal Turlure, Jean-Pierre Marolleau, Oumedaly Reman, Claude Gardin, Maud Victor, Sébastien Maury, Philippe Rousselot, Jean-Valère Malfuson, Odile Maarek, Marie-Thérèse Daniel, Pierre Fenaux, Laurent Degos, Christine Chomienne, Sylvie Chevret, Hervé Dombret

**Affiliations:** ^1^ Département d'Hématologie, Hôpital Saint-Louis, Assistance Publique – Hôpitaux de Paris (AP-HP), and Université Denis Diderot - Paris 7, EA 3518, Institut Universitaire d'Hématologie (IUH), Paris; ^2^ UMR-S-940, Université Denis Diderot-Paris 7, IUH, Paris; ^3^ Département d'Hématologie, Hôpital Purpan, Toulouse; ^4^ UMR-S-707, Université Pierre et Marie Curie – Paris 6, Hôpital Saint-Antoine, AP-HP, Paris; ^5^ Département d'Hématologie, Hôpital Dupuytren, Limoges; ^6^ Département d'Hématologie, Hôpital Nord, Amiens; ^7^ Département d'Hématologie, Centre Hospitalier Universitaire, Caen; ^8^ Département d'Hématologie, Hôpital Avicenne, AP-HP, Bobigny; ^9^ Département d'Hématologie, Hôpital Henri Mondor, AP-HP, Créteil; ^10^ Département d'Hématologie, Hôpital Mignot, Versailles; ^11^ Département d'Hématologie, Hôpital Percy, Clamart; ^12^ Département de Biostatistiques et d'Informatique Médicale, Hôpital Saint-Louis, AP-HP, Paris, France

**Keywords:** acute myeloid leukemia, azacitidine, valproic acid, ATRA, FZD9 methylation

## Abstract

In this Phase 2 study, we evaluated the efficacy of combination of 5-azacitidine (AZA), valproic acid (VPA), and all-trans retinoic acid (ATRA) in patients with high-risk acute myeloid leukemia (AML) or myelodysplastic syndrome (MDS). Treatment consisted of six cycles of AZA and VPA for 7 days, followed by ATRA for 21 days. Sixty-five patients were enrolled (median age, 72 years; 55 AML including 13 relapsed/refractory patients, 10 MDS; 30 unfavorable karyotypes). Best responses included 14 CR and 3 PR (26%), 75% of the responders and 36% of the non-responders achieving an erythroid response. Median overall survival (OS) was 12.4 months. Untreated patients had a longer OS than relapsed/refractory patients. In patients who fulfilled the 6 planned cycles, OS did not appear to depend on CR/PR achievement, suggesting that stable disease while on-treatment would be a surrogate for survival with this approach. During therapy, early platelet response and demethylation of the *FZD9*, *ALOX12*, *HPN*, and *CALCA* genes were associated with clinical response. Finally, there was no evidence for the restoration of an ATRA-induced differentiation during therapy.

Epigenetic modulation deserves prospective comparisons to conventional care in patients with high-risk AML, at least in those presenting previously untreated disease and low blast count.

## INTRODUCTION

Chromatine remodeling through DNA demethylation has been investigated for many years as a potential anticancer therapeutic approach [1]. Two DNA methyltransferase inhibitors, 5-azacytidine (AZA) and decitabine, have demonstrated clinical activity as single agents in patients with MDS/AML [2-7]. Both drugs are registered in the US to treat patients with MDS. Based on a large confirmatory study [8], AZA has recently obtained an EMEA approval to treat patients with high-risk MDS and AML until 30% marrow blasts, as the survival benefit over conventional care regimens was also observed in the subgroup of patients with low bone marrow blast count AML [9].

Another way to modulate the epigenetic chromatine structure is to use histone deacetylase (HDAC) inhibitors. It has been shown for instance that the resistance to all-trans retinoic acid (ATRA) observed in acute promyelocytic leukemia (APL) cells carrying the variant PLZF-RARA fusion protein may be abrogated by HDAC inhibitors (HDACi) such as trichostatin A or sodium phenylbutyrate *in vitro* or *in vivo* [10-11]. Valproic acid (VPA) has been demonstrated as belonging to the HDACi family and, interestingly, combined VPA/ATRA treatment may also induce hematological responses in patients with non-APL AML or MDS [12-14].

In this Phase 2 study, we thus evaluated the efficacy of AZA/VPA/ATRA combination in patients with high-risk AML or MDS. Focusing on differential analysis between responders and non-responders, DNA methylation profile analysis confirmed that epigenetic modulation occurs *in vivo* and may be linked to drug efficacy.

## RESULTS

### Patients

A total of 65 patients (median age, 72 years; ranging from 50 to 87) entered the study. Patient characteristics are shown in **Table 1**. The numbers of patients were 42, 13, and 10 in eligibility subset 1, 2, and 3, respectively. All patients with refractory/relapsed AML had previously received intensive chemotherapy. The proportion of patients with unfavorable cytogenetics was high (52%); no patient had favorable core binding factor AML. Median WBC and percentage of marrow blasts were low, suggesting that a selection of patients with slowly progressing disease may have occurred. At the reference date of analysis, the median follow-up was 16.3 months, ranging from 14 to 28.

**Table 1:** Patient characteristics

Patients	N= 65
Male/female	38/27
Median age (Q1-Q3)	72 Years (70-77)
>70 Years	48 (74%)
Performance status (N,%)	
0	20 (31%)
1	37 (58%)
>1	7 (11%)
NA	1
Median WBC count (Q1-Q3)	2.3 × 10^9^/L (1.6-4.7)
Median platelet count (Q1-Q3)	43 × 10^9^/L (19-73)
Median marrow blast percentage (Q1-Q3)	31% (20-53)
Disease subsets (N, %)	
Previously untreated AML	42 (65%)
Relapsed/refractory AML	13 (20%)
High-risk MDS	10 (15%)
Cytogenetics (N, %)	
Standard	28 (48%)
Unfavorable *	30 (52%)
NA	7

* unfavorable karyotypes were defined as −7, del(7q), −5, 3q abnormality, or complex (5 anomalies or more); NA: not available.

### Compliance and response

Details on compliance to therapy, as well as reasons for treatment discontinuation, are given in **Table 2**. Three patients never started the treatment (1 consent withdrawal, 2 very early deaths). The main causes of treatment discontinuation were disease progression (8 patients) and death (15 patients). Death was related to or concomitant of disease progression in 5 of these 15 patients. Nine patients died from infection and another one died from myocardial infarction. Only 3 patients withdrew their consent during the study time.

**Table 2:** Treatment compliance and responses

After cycle	Patients	CR	PR	Stable	Progression	NA*	Reasons for treatment discontinuation (N)			
N	N	N(%)	N(%)	N(%)	N(%)		Patient decision	Disease progression	Severe toxicity	Death
0	65	-	-	-	-	-	1	0	0	2
1	62	0 (0%)	0 (0%)	38 (61%)	10 (16%0	14 (23%)	1	1	2	7
2	51	-	-	-	-		0	0	3	3
3	45	8 (18%)	3 (7%)	26 (57%)	8 (18%)	0 (0%)	0	4	0	1
4	40	-	-	-	-		1	1	0	2
5	36	-	-	-	-		0	2	0	0
6	34	13 (38%)	2 (6%)	14 (41%)	5 (15%)	0 (0%)	-	-	-	-

* response was not recorded after cycle 1 in 14 patients still on study.

No complete (CR) or partial (PR) remission was observed after the first cycle. Among the 45 patients who received the first 3 cycles, 8 (18%) were in CR and 3 (7%) in PR at that time (**Table 2**). Among the 34 patients who received the six planned cycles, 13 (38%) achieved CR and 2 (6%) achieved PR (**Table 2**). Best responses were, however, 14 CR and 3 PR (26%), as some patients lost their response between cycle 3 and 6. Responses were seen within the three eligibility subsets: 11 CR and 2 PR in newly-diagnosed elderly patients, 2 CR and 1 PR in relapsed/refractory patients, and 1 CR in high-risk MDS patients (P= 0.47). Among the 15 patients alive in response after cycle 6, 14 patients received further maintenance treatments with AZA alone (7 patients) or low-dose cytarabine (7 patients). At the 6-cycle evaluation time, the cumulative incidence of CR + PR was 27% (95% CI: 26-28%), while that of treatment discontinuation was 15.5% (95% CI: 14.5-16.5%) and that of death before response was 31% (95% CI: 30-32%).

**Fig. 1:**
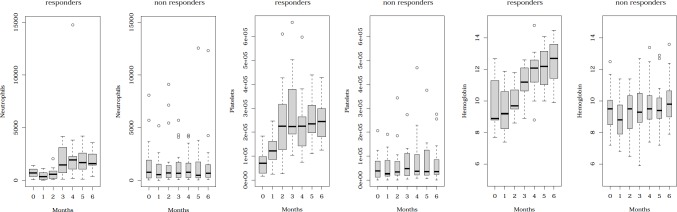
Kinetics of peripheral blood neutrophil count, platelet count, and hemoglobin level during the treatment period. The evolution of peripheral blood neutrophil count (/mm^3^), platelet count (/mm^3^), and hemoglobin level (g/dL) is shown in the 34 patients who may receive the planned 6 treatment cycles, according to the response (15 CR + PR patients versus 19 non-responding patients).

Median PB neutrophil count, platelet count, and Hb level observed during therapy in the 34 patients who received the 6 planned treatment cycles are shown in **Figure 1** according to the 6-month response (15 responding and 19 non-responding patients). As indicated, platelet count improvement was the earliest feature observed in responding patients. Stable PB counts or partial improvements were, however, observed in non-responding patients. With respect to erythroid response, 75% of the responders and 36% of the non-responders met criteria for ER after 6 cycles. Erythroid response was never observed after the first cycle, but 10% of the responders and 12.5% of the non-responders reached it after 3 cycles.

Factors usually associated with the outcome of AML/MDS patients (age, PS, cytogenetics, and disease status) predicted death before response during therapy. These factors, including cytogenetics, did not, however, predict the response. The only factor associated with a lower response incidence was a low platelet count (18% CR + PR in the 24 patients with initial platelet count < 50.10^9^/L). In addition, early platelet recovery after the first cycle was significantly associated with a higher response rate (P< 0.001 for platelet count as a continuous variable). Response incidence reached 57% in patients with a platelet count of 100 × 10^9^/L or more after the first cycle compared to 12% in other patients (P= 0.001).

### Toxicity

Main adverse events are reported in **Table 3**. If one except fatigue, infections, and hemorrhages usually observed in AML patients, the toxicities observed may readily be related to one of the three drugs administered. Somnolence and confusion related to VPA led to amend the starting VPA dose after the first 11 patients. Gastro-intestinal events were probably related to AZA, as were pain at the AZA injection sites and fatigue. Mucosal dryness is a well-known side effect of ATRA. In the 34 patients who received the 6 cycles, re-hospitalization rate was 29%, 38%, 23%, 18%, 15%, and 15% after cycle 1 to 6, respectively.

**Table 3:** Adverse events

Events	Number of events	Cycle of occurrence (Mean ± SD)
Confusion	33	1.7 ± 1.4
Fatigue	20	2.0 ± 1.5
Somnolence	12	1.3 ± 1.4
Constipation	13	1.0 ± 1.1
Nausea / Vomiting	10	2.5 ± 1.7
Hemorrhage	13	2.0 ± 1.4
SC puncture site reaction	9	1.7 ± 1.9
Mucosa dryness	8	1.7 ± 2.0
Infection		
All events	76	2.0 ± 3.3
Pneumonia	13	-
Septicemia	17	-
Fungal infection *	2	-

* two invasive *Aspergillus sp.* infections.

### Survival

**Fig. 2:**
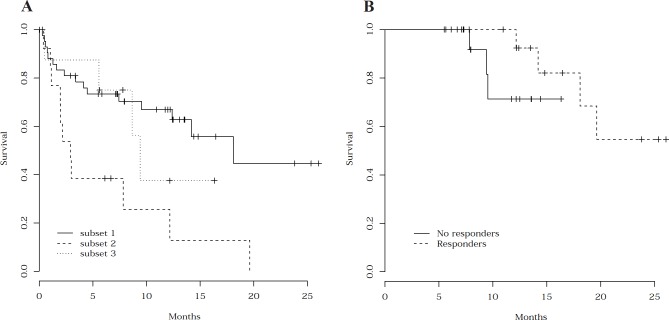
Overall survival **(A)** OS from study inclusion according to the three eligibility subsets. OS was significantly shorter in patients relapsing after prior intensive chemotherapy (subset 2) than in naïve patients with either AML (subset 1) or high-risk MDS (subset 3) (P= 0.0024). **(B)** OS following the 6-month evaluation according to the response observed at 6 months. In the 34 patients who received the 6 planned cycles, OS after the 6-month evaluation was not significantly different between responding (N= 15) and non-responding (N= 19) patients.

**Figure 2A** shows OS in the 65 enrolled patients, according to the eligibility subsets. Median OS was 12.4 months for the whole patient cohort, with a significant difference between naïve AML/MDS patients and relapsing patients (18.1 *versus* 2.9 months; P= 0.0024). Median OS of the 17 responders was 19.6 months. In the 34 patients who received the 6 planned cycles, OS from 6-month evaluation was not significantly different between responding and non-responding patients (**Figure 2B**), meaning that the survival of patients who were still on therapy at that time did not seem to be significantly influenced by CR or PR obtention. Factors previously shown to be associated with death during therapy (age, PS, cytogenetics, and disease status) were also predictive of OS. Based on multivariable Cox model, only PS and unfavorable cytogenetics were associated with a shorter OS.

### Genome methylation

Baseline methylation profiles were available in 28 patients. After excluding sexual chromosome-related sites, 1421 regulatory regions were analyzed. Methylation profiles were bimodal with 454 sites with a median methylation of 50% or more (32%). Methylation profiles were relatively similar among patients and unsupervised clustering did not allow identifying subgroups with different patterns, even when focusing on hypermethylated loci (data not shown). Similarly, supervised clustering did not allow to allocate specific signatures to various patient subsets defined by age, gender, PS, WBC, platelet count, percentage of marrow blasts, disease status, cytogenetics, or even response to therapy. After adjustment for multiple testing, only one site showed significant correlation with WBC (*PMP22*, P=0.04) and another with marrow blast percentage (*TFDP1*, P=0.01), but no site was significantly differently methylated at baseline between responders and non-responders.

Eight patients had an additional methylation profile obtained after 1 to 3 treatment cycles. Four of these patients achieved a response at 6-month evaluation (2 CR and 2 PR), while four patients did not respond. **Table 4A** shows the sites that were strongly differentially demethylated during therapy between responders and non-responders. All these genes (*FZD9*, *ALOX12*, *HPN*, and *CALCA*) were hypermethylated at baseline and demethylated during therapy in responding patients, even if not reaching the statistical significance level after adjustment for multiple testing.

Table 4Methylation changes according to the response to therapy

**Table:** **(A)** Regulatory regions associated with the largest differences in demethylation between responding and non-responding patients during the therapy.

	Non-responding patients* (n=4)			Responding patients** (n=4)		
Methylation level	Before Tx	After Tx	Δ	Before Tx	After Tx	Δ
*FZD9*	75%	77%	2%	57%	34%	-23%
*ALOX12*	88%	90%	2%	77%	55%	-22%
*HPN*	76%	73%	-3%	71%	45%	-26%
*CALCA*	43%	43%	0%	60%	37%	-23%

**Table:** **(B)** Methylation changes observed at RA target gene regulatory regions.

	Non-responding patients* (n=4)			Responding patients** (n=4)		
Methylation level	Before Tx	After Tx	Δ	Before Tx	After Tx	Δ
*RARA*	20%	20%	0%	19%	14%	-5%
*RARA*	43%	43%	0%	36%	46%	10%
*RARA*	47%	51%	4%	47%	40%	-7%
*RARB*	4%	4%	0%	4%	5%	1%
RARB	13%	9%	-4%	19%	12%	-7%
*CDKN1A*	8%	7%	-1%	8%	10%	2%
*CDKN1A*	4%	5%	1%	6%	5%	-1%
*RBP1*	6%	7%	1%	7%	8%	1%
*RBP1*	7%	7%	0%	13%	4%	-9%
*RBP1*	22%	17%	-5%	40%	26%	-14%
*ETS1*	25%	23%	-2%	31%	33%	2%
*ETS1*	3%	4%	1%	4%	4%	0%

* none of these four patients achieved CR or PR at 6-month evaluation;

** 2 patients achieved CR and 2 patients achieved PR at 6-month evaluation;

Δ indicates the decrease in gene promoter methylation level observed during therapy (Tx).

### Induction of differentiation

There was no evidence supporting the hypothesis of ATRA-induced differentiation restoration, even in responding patients. Morphological features suggesting *in vivo* differentiation of AML blasts, reminiscent of those observed in APL patients under ATRA treatment, were observed in one CR patient only. Unfortunately, this patient did not have cytogenetic aberration that could have been monitored to further support this hypothesis. No clinical symptoms suggesting any differentiation syndrome were noted. In addition, no clear demethylation was observed at various retinoic acid target gene loci during therapy. Retinoic acid receptor alpha (*RARA*) and beta (*RARB*) gene promoters were rather hypomethylated at baseline and no significant change in their methylation levels was observed during therapy (**Table 4B**). Although demethylation of the two *RBP1* loci appeared slightly superior in responding than in non-responding patients, no clear differences between responding and non-responding patients were observed for three other *CDKN1A*, *RBP1*, and *ETS1* retinoic acid target genes.

## DISCUSSION

We report here a 26% response rate including 22% CR associated with AZA/VPA/ATRA treatment in patients with high-risk AML or MDS. Even if selected, the 65 patients enrolled were clearly at high risk of treatment failure: their median age was over 70 years and, more importantly, half of them had very unfavorable cytogenetic features. Similar response rates have been reported using various combination of the same drugs, even if using different schedules [18-19]. In the first study from the M.D. Anderson Cancer Center (MDACC) which included a much shorter ATRA exposure (5 days per cycle, from day 3 to 7), the overall CR rate was 23% [18]. As in the present study, responses were observed across the different risk subsets including unfavorable cytogenetics. In the British study which added theophylline to AZA/VPA/ATRA as a fourth pro-differentiating agent, the overall CR/CRi/PR rate was 33% [19].

It appears thus that ATRA does not add any significant clinical benefit to treatments based on epigenetic modifying drugs. Various 2-drug or even 1-drug regimens with DNA methyltransferase inhibitors and/or HDACi have anti-AML efficacy [2-10, 20] and response rates observed in these three ATRA-containing studies are not clearly superior. Furthermore, similar response rates were observed when using either 5 days of ATRA administration per treatment cycle, as in the MDACC study [8], or 21 days, as in the present study. In addition, we were unable to provide observations supporting a significant differentiating effect of ATRA when combined to AZA/VPA in such non-APL patients.

Whether the superiority of AZA/HDACi combinations over the single-agent AZA therapy remains questionable, as no controlled study has yet prospectively addressed this issue. Some *in vitro* data are in favor of additive or synergistic effects when adding VPA to a DNA methyltransferase inhibitor [21]. The single randomized Phase 2 study which has addressed this issue in MDS/AML patients has used decitabine but not AZA, and reported only marginal response rate and time to response improvements without impact on survival [22]. Of interest, combinations of azacitidine with newer and potentially less toxic HDACi, such as vorinostat or entinostat, have also been reported as associated with good response rates [23-24].

The present study provides two additional important clinical observations. The first one is the prognostic value of a rapid platelet count recovery, often observed as soon as during the first cycle in responders and suggesting a direct effect of any component of the treatment on the megakaryocytic lineage. The second one is the apparent good outcome of patients who did not reach CR or PR but may receive the whole planned treatment. Similar results were recently reported in high-risk MDS patients [8]. This confers an unusual feature to AZA, which appears as a drug able to significantly prolong survival even in the absence of hematological response.

Maybe due to the low number of patients studied, we were unable to find specific methylation profile signatures that distinguished responders from non-responders. We nonetheless identified a set of four genes that were markedly demethylated in responding patients, while unchanged in non-responding patients. Interestingly, these four genes (*FZD9*, *ALOX12*, *HPN*, and *CALCA*) have been recently reported as aberrantly methylated in MDS/AML patients during the progression from MDS to AML [25]. Among these genes, *FZD9* is a receptor of Wnt and a putative tumor suppressor gene located on chromosome 7. It is thus tempting to consider that patients responding to epigenetic modulation might somehow “return” to a less advanced pre-leukemic state. In the British study mentioned above [19], it was observed that some patients may reach CR with good restoration of marrow progenitor quantity and quality, while still harboring persistent malignant stem cells [19]. If confirmed, these observations should encourage further approaches to maintain long lasting responses in these patients, including stem cell transplantation, immunomodulators, or even vaccination.

In conclusion, as shown by the median OS reported here, epigenetic modulation deserves now direct randomized comparisons to other conventional care regimens in older patients with AML, at least in those presenting previously untreated disease and relatively low blast count. Identification of reliable biomarkers that could predict response to these new therapies remains an important issue to select patients most likely to derive a prolonged benefit from these treatments.

## PATIENTS AND METHODS

### Study design and treatments

The study (ClinicalTrials.gov ID, NCT00339196) was approved by the Ethics Committee of the Pitié-Salpêtrière Hospital, sponsored by the Délegation à la Recherche Clinique (DRRC ID, P050202), and conducted between 2006 and 2007 in 9 French centers. All patients signed informed consent in accordance to the Declaration of Helsinki. Patients were planned to receive six monthly cycles of AZA/VPA/ATRA. AZA was given subcutaneously at 75 mg/m2/d in combination with oral VPA at 35 to 50 mg/kg/d, both for 7 days (day 1 to 7). ATRA was then given orally at 45 mg/m2/d from day 8 to 28. Patients were admitted in hospitals for cycle 1, while they were treated on an outpatient basis for cycles 2-6, which were repeated whatever peripheral blood (PB) counts but not earlier than every 4 weeks. At the onset of the study, VPA was started at 50 mg/kg/d in all patients. After enrolment of the first 11 patients, excessive VPA-induced neurological toxicity observed in some patients led the independent Data Safety and Monitoring Committee to recommend a 35 mg/kg/d VPA starting dose for the first cycle, that could then be increased to 50 mg/kg/d for subsequent cycles if clinically tolerated. Supportive care measures including antibiotics, antiemetics, and growth factors were allowed if clinically indicated and according to institutional guidelines. After the 6th cycle, responding patients could receive further maintenance treatments.

### Eligibility criteria

High-risk AML was defined as newly-diagnosed previously untreated AML in patients aged 70 years or more unlikely to benefit from standard intensive chemotherapy (subset 1) or relapsed/refractory AML in patients with a first CR duration < 18 months and/or post-MDS AML (subset 2). High-risk MDS was defined as refractory anemia with excess blasts or refractory anemia with excess blasts in transformation with an intermediate-2 or high International Prognostic Scoring System (IPSS) score [15] (subset 3). Eligibility criteria also included an ECOG performance status (PS) score not higher than 3 and adequate hepatic and renal functions. Patients must have been off chemotherapy or other investigational therapy for at least 4 weeks prior study entry and not previously treated with AZA, VPA, or ATRA. Patients with APL or central nervous system leukemic involvement, or patients with active or uncontrolled infection were excluded, as well as those with a white blood cell count (WBC) ≥ 30 × 10^9^/L. Pre-treatment with hydoxyurea was allowed, but had to be interrupted at least 48 hours prior to study entry.

### Cytogenetic and response classification

Unfavorable cytogenetics was defined as −7, del(7q), −5, 3q abnormality, or complex (5 anomalies or more). All other karyotypes were classified in a standard-risk group. Bone marrow and PB response was assessed after cycle 1, 3, and 6, and classified according to the International Working Group (IWG) AML criteria [16]. Erythroid response (ER) was defined according to IWG MDS criteria [17].

### Genome methylation assay

Marrow samples were obtained at baseline in 28 patients and under therapy in 8 patients. Mononuclear cells were isolated using Ficoll density gradient centrifugation (Eurobio, Les Ulis, France). Genomic DNA was prepared using the DNA Blood Mini Kit (Qiagen, Courtaboeuf, France) and then subjected to sodium bisulfite conversion using the EZ DNA Methylation Kit (Zymo Reseach, Orange, CA). DNA methylation profiles were determined using the GoldenGate Methylation Cancer Panel I (Illumina, San Diego, CA). Briefly, bisulfite-converted DNAs are biotinylated, hybridized to query oligos, and washed. The hybridized oligos are then extended and ligated to create amplifiable templates. The PCR that follows uses fluorescently labeled universal PCR primers. The resulting PCR products were hybridized to a bead array at sites bearing complementary address sequences. These hybridized targets contained a fluorescent label that tagged methylated or unmethylated sequences at a given locus. Methylation status of the interrogated CpG sites was determined by comparing the ratio of the fluorescent signal from the methylated allele to the sum from the fluorescent signals of both methylated and unmethylated alleles yielding a percentage of methylation at each site. The GoldenGate Methylation Cancer Panel I assay probes 1505 CpG sites from the 5' regulatory regions of 807 cancer-associated genes on 23 chromosomes.

### Statistical methods

Analysis was performed on the intent-to-treat basis. Cumulative incidences of response were estimated considering treatment discontinuation and death before response as competing risks and compared with the Gray test. Overall survival (OS) was estimated by the Kaplan-Meier method, with reference date of November 15th, 2008. Survival comparisons were based on Cox proportional hazards models. The following factors were analyzed for their prognostic value: gender, age, PS, WBC, platelet counts, marrow blast percentage, cytogenetics, and relapsed/refractory status. All statistical tests were two-sided. For DNA methylation assay analysis, the 84 regulatory regions located on sexual chromosomes were excluded. The raw profiles were normalized using quantile normalization. Unsupervised clustering was applied using the euclidean distance. Initial methylation profiles were correlated with baseline characteristics and response at 6 months using the Spearman correlation coefficient. Changes in methylation levels were investigated in a subset of patients for whom a second methylation profile was available after initiation of treatment. Multiple testing correction was applied using the Bonferroni single step method for P values. All analyses were performed on SAS 9.1 (SAS, Cary, NC) and R 2.8.0 software packages (http://www.R-project.org).
